# Grounding digital mental health and wellbeing platform development in a theory of change: a convergent mixed methods approach

**DOI:** 10.3389/fpsyt.2025.1637861

**Published:** 2025-10-28

**Authors:** Emily P. Cowling, Regina Misch, Georgia Sugarman, Samaryah Sammut, Terry Hanley, Roy Sugarman, Lynne Green, Patrick Johnston, Tamara Ramos, Brian Rock, Hannah Wilson, Louisa Salhi

**Affiliations:** ^1^ Kooth Digital Health, Chicago, IL, United States; ^2^ Kooth Digital Health, London, United Kingdom; ^3^ Manchester Institute of Education, University of Manchester, Manchester, United Kingdom; ^4^ Medical School, Medical Education, University of New South Wales, Sydney, NSW, Australia

**Keywords:** adolescent, development framework, digital intervention, early intervention, user-centered, psychological flexibility, logic model

## Abstract

**Background:**

Mental health concerns among children and young people are rising, yet only a fraction receive the care they need. Digital mental health solutions can help bridge this gap, and recent years have seen a rapid proliferation of mental health applications. However, many lack a clear framework detailing how their activities lead to meaningful outcomes. A theory of change articulates these pathways, informing the development, implementation and evaluation of such technologies. Using a case illustration, this paper presents the practical application of developing a theory of change for a digital mental health intervention.

**Methods:**

In 2024, Kooth Digital Health launched a digital mental health platform, Soluna, to all 13-to-25 year olds in California. Its development was grounded in a theory of change, co-created with youth, service staff, and external experts. A convergent mixed methods design was utilized to develop the theory of change across three phases of work via a digital diary, workshop series and express media survey. Using an iterative approach, data analyzed from one phase influenced the next. Qualitative data were analyzed using rapid deductive analysis and quantitative data were summarized using descriptive statistics.

**Results:**

In the insight generation phase, 50 youth completed a digital diary while using the proof-of-concept application. Findings suggested youth sought autonomy, relatedness, and self-efficacy, central to their engagement and desired outcomes. The framework development phase engaged 18 service staff and two external experts via workshops to define the theory of change, mapping the platform’s features to intended outcomes. In the dissemination design phase, 12 youth provided survey feedback on the theory of change, validating that its key concepts resonated with them and informing content clarity revisions.

**Conclusions:**

The methodology led to recommendations for developing a theory of change for industry applications: (i) consult diverse stakeholders, including youth, throughout, (ii) utilize a mixed methods design to triangulate data, (iii) leverage interactive methods to facilitate data collection, (iv) be flexible in the approach, and (v) engage advocates. Applying these methods can help developers and researchers design, implement and evaluate digital mental health interventions that are user-centered and effective.

## 1 Introduction

The prevalence of mental health concerns among children and young people continues to rise, calling for scalable solutions to meet the growing demand for mental health support. According to the World Health Organization (WHO), one in seven youth aged 10 to 19 experiences a mental health disorder, and suicide is the fourth leading cause of death among young people aged 15 to 29 years (1). Mental Health America estimates that 46% of Americans will experience a diagnosable mental health condition at some point in their lifetime, with half of these conditions emerging by the age of 14 (2). Despite the growing need for mental health treatment, only a fraction of people with mental health conditions receive the care they need. In the US, over 60% of youth with major depression go without treatment (2), and in California, this statistic rises to 64.5% (3).

This need, further accelerated by the necessary shelter-in-place measures of the COVID-19 pandemic, has given rise to the proliferation of digital mental health interventions (4, 5). In light of this, there is an urgent need for a structured approach to developing digital solutions to ensure these tools effectively serve their intended purpose and produce a positive impact. While there is a saturation of applications being marketed to enhance mental wellbeing, many lack a clear framework that connects their application features and activities with meaningful outcomes for the users (6, 7).

**Figure 1:**
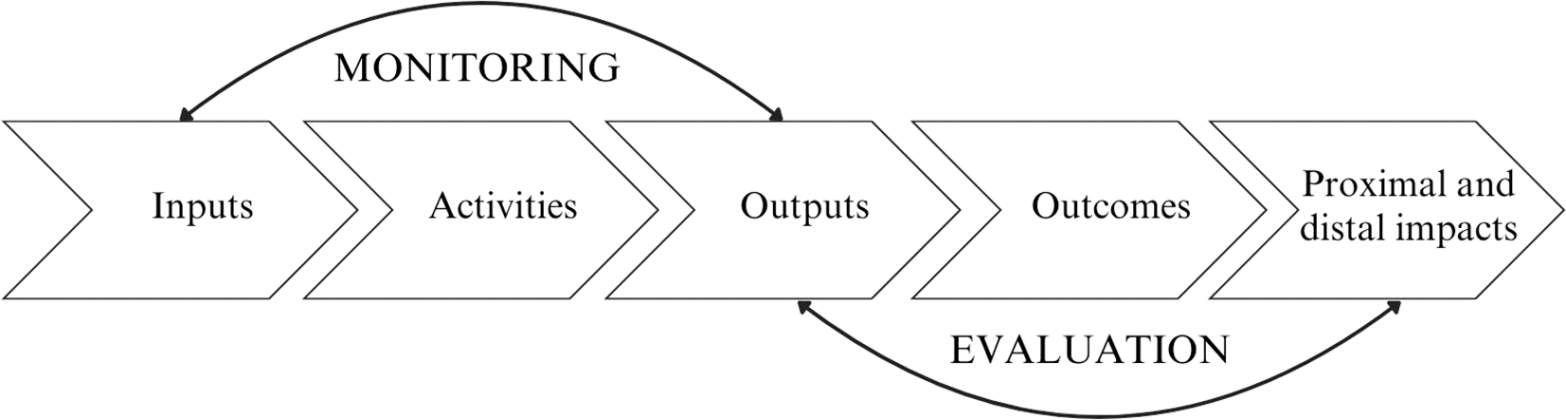
The structure of a theory of change framework (adapted from[11]).

A theory of change articulates how a strategy or intervention will bring about change at an individual, organizational, or societal level by clarifying how activities and actions will lead to desired outcomes, thus fostering transparency, accountability, and evidence-informed decision-making (8). As a logic model, a theory of change is a comprehensive framework that illustrates the pathways for how change is expected to occur, linking the intervention’s inputs and activities to desired impacts, while detailing the assumptions and mechanisms that underpin this process (9, 10). This provides an integrated approach to designing, implementing, and evaluating the intervention (11) (**Figure 1**).

A theory of change is often used in developing social and behavioral programs and strategies to keep goals in focus and develop meaningful measurements of impact (8). In the context of digital mental health, a theory of change can provide a structured framework that informs platform development, guides ongoing optimization to implementation, and establishes the foundation for a robust evaluation framework. However, digital mental health applications are often seen primarily as products rather than interventions, thus relying on metrics such as retention and engagement as success indicators, at the cost of overlooking process, system, and outcome indicators that are essential to long-term outcomes (12). Without a framework that matches activities to desired outputs, including the clear mechanisms of behavior change, applications risk being developed without a clear understanding of what works, for whom, and in what context (7, 13, 14). It has been acknowledged that the mechanism of change frameworks for digital health interventions are lacking and that frameworks specific to these types of interventions are needed as they pose unique challenges that differ from non-digital interventions (15).

This paper presents the practical application of developing a theory of change for a digital mental health intervention. Using a case illustration from Kooth Digital Health, the paper demonstrates how such a framework helps solidify the pathways through which the intervention achieves its goals. By drawing on clinical evidence, user preferences, and existing literature, the paper highlights an iterative, evidence-based approach that informs product development, stakeholder engagement, and future evaluation, while bridging academic theories with real-world industry practice.

## 2 Materials and methods

### 2.1 Description of the digital mental health and wellbeing platform

Kooth Digital Health provides web-based counseling and support services for youth and adults across the United Kingdom and the United States. As part of the Child and Youth Behavioral Health Initiative (CYBHI) to transform behavioral health services for young people aged 13 to 25 in California, Kooth developed a new digital platform, Soluna, that launched on January 1, 2024. Soluna, offered through the CalHOPE program, provides free access to various support options, including one-to-one coaching, behavioral health content and wellbeing activities, a pre-moderated peer community, and connection to other services and supports through care navigation. Soluna takes a novel approach to mental health and wellbeing support by transitioning from a traditional diagnostic model to a preventative, skills-based framework that facilitates long-term behavioral change. This aligns with the service’s humanistic value base (16, 17), which emphasizes a strengths-based, integrative approach to support that prioritizes agency of users (18).

To build a user-centered, effective, and scalable product, the platform’s development was grounded in a theory of change. This case illustration describes the iterative process by which the theory of change for the platform was created through stakeholder engagement.

### 2.2 Stakeholder engagement

Three key stakeholder groups were consulted in the development of the theory of change: youth from the platform’s target population, cross-functional staff from the digital mental health service, and external academic and industry experts. Youth provided insights into their wants and needs, ensuring that the platform was designed to be relevant, engaging, and responsive to the challenges youth are facing. Service staff members contributed significant knowledge about the way young people use and benefit from digital mental health services. External experts offered evidence-based perspectives, integrating established theories and frameworks to develop and validate the theory of change, while also providing practical insights to ensure the framework’s applicability in its real-world context.

### 2.3 Design

**Figure 2:**
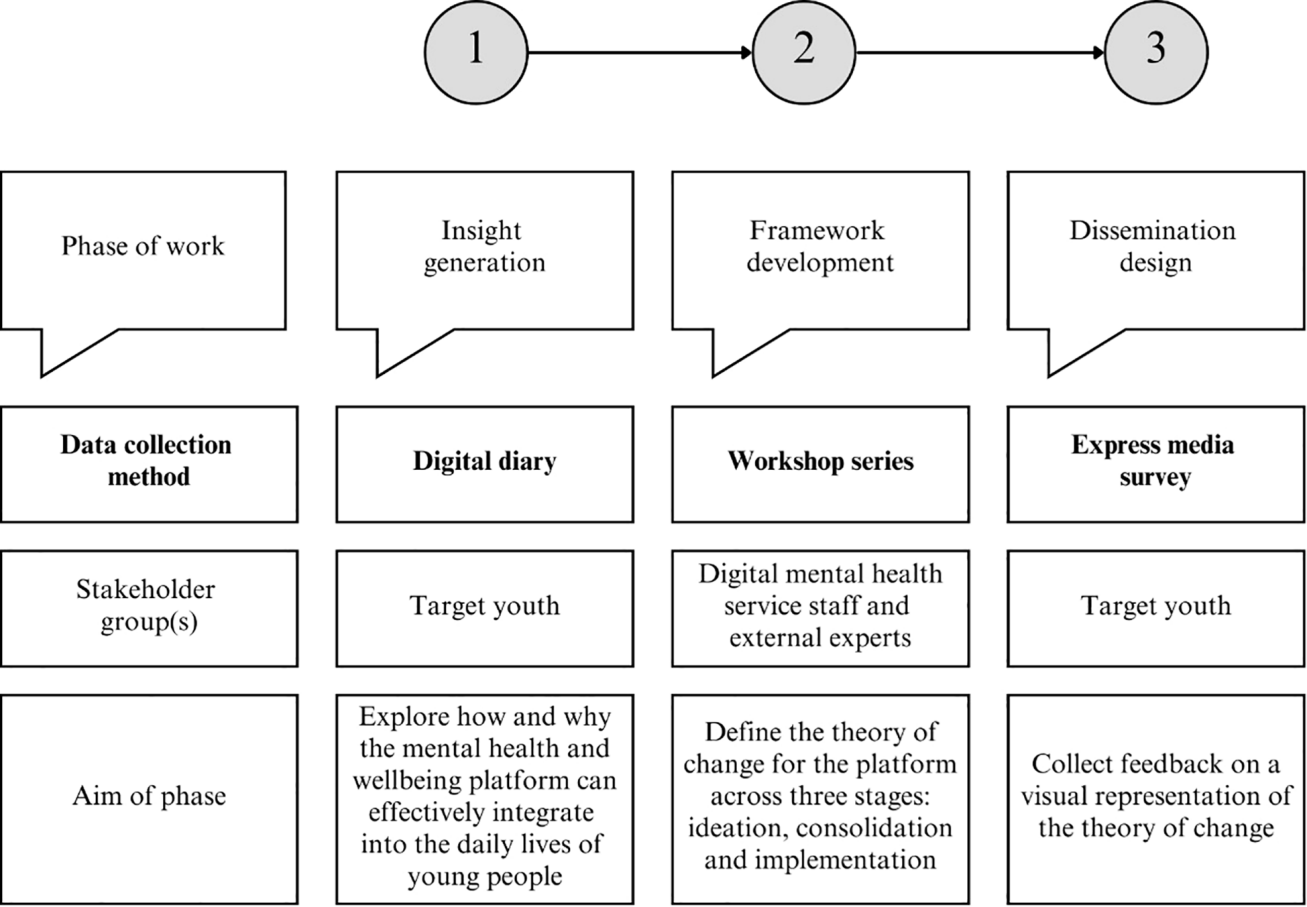
A flow chart outlining the three phases of work, data collection methods, consulted stakeholder groups and the aim of each phase. Qualitative data were analyzed using rapid deductive analysis, while quantitative data were examined using descriptive statistics.

A convergent mixed methods design was implemented, combining qualitative and quantitative data collection methods during a similar timeframe. Using an iterative approach during this timeframe, data collected and analyzed were integrated at the design level, meaning that the results from one phase influenced the data collection procedures of another (19). The work was organized across three interconnected phases, which emerged as the work progressed: (1) insight generation, where youth used a digital diary to provide feedback on the proof-of-concept application, (2) framework development, which involved a workshop series with service staff and external experts to collaboratively define the theory of change (logic model), and (3) dissemination design, where an express media survey was used to validate a visual representation of the theory of change based on youth feedback (**Figure 2**).

### 2.4 Data collection

For the three phases of work, distinct data collection methods were employed, tailored to the consulted stakeholder group(s) and specific aim, as shown in **Figure 2**. The following section details the methods used in each phase.

#### 2.4.1 Insight generation phase

For the insight generation phase, a digital diary, referred to as a ‘diary mission’, was conducted on the online platform dscout (20) to understand how and why youth might integrate a mental health and wellbeing platform into their daily lives. This phase was conducted as part of the grant proposal for CYBHI to provide youth feedback on the proof-of-concept application (hereafter referred to as the proof-of-concept app). Young people expressed their interest in the diary mission by completing the public screener that was made available to individuals in the dscout pool aged 13 to 25 in California, representing the target population of the platform. Eligible youth were then invited to the eight-day diary mission, with the sampling method using oversampling to ensure diverse representation, including a majority of young people who identify as Black, Indigenous, and people of color (BIPOC) and with lived experience seeking mental health support.

For the mission, youth were given access to the proof-of-concept app to use as they liked for eight days. As they engaged with the app, youth were asked to share their experiences using the built-in diary entry tool on dscout, designed for safe, engaging and accessible participation (20). This tool enabled youth to document their experience in real-time and at their own pace, without staff present. Media-rich feedback was collected through video recordings in which young people followed a ‘think-aloud’ protocol (21) to verbalize their thoughts while interacting with the app. The dscout artificial intelligence analysis tool (20) produced written transcripts for the video recordings. Feedback was also gathered using qualitative open-ended questions, as well as quantitative five-point scale, item-ranking and multiple-choice questions. At the close of the mission, youth were compensated $100 for their time, in accordance with dscout’s policy (20).

#### 2.4.2 Framework development phase

**Figure 3:**
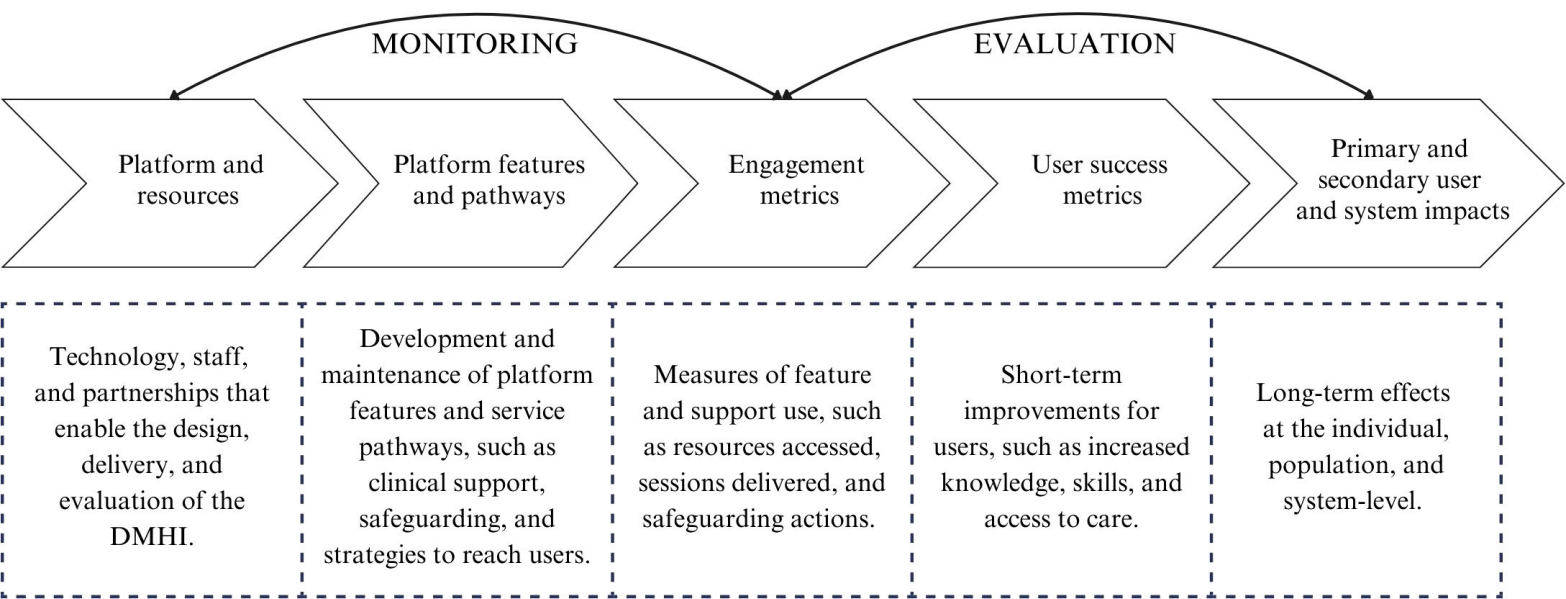
Industry adaptation of a theory of change framework for a digital mental health intervention (adapted from[11]).

A workshop series was carried out to define the theory of change framework. Adapted to an industry setting, the framework would illustrate the pathways for how change is expected to occur by linking the platform’s features to the intended user success metrics and primary impact on user wellbeing (**Figure 3**).

In this phase, the first step was to identify a psychosocial approach to guide the workshop discussions. A behavioral change model was selected, emphasizing the processes and factors that influence an individual’s decision to adopt new behaviors. Existing models, such as the Behavior Change Wheel and the embedded COM-B (Capability, Opportunity, Motivation and Behavior) model (22) were reviewed to inform the initial workshop.

The workshop series involved 20 individual stakeholders (18 cross-functional staff members from the digital mental health service and two academic and industry experts). Workshops were scheduled at varying times and conducted either in-person or online via Zoom (23) to accommodate stakeholders in the United Kingdom, United States of America, and Australia.

Workshops utilized the visual workspace, Miro (24). A board was set up on Miro, serving as a central hub for stakeholders throughout the series. The board provided key information about the series, including (1) an introduction to a theory of change to guide project developers, (2) a clear outline of the workshop series, specifying its aims and boundaries, (3) a designated ‘parking lot’ space for questions that required further exploration but could not be addressed in the moment, and (4) dedicated areas for all workshop activities.

Each workshop was designed, facilitated, and analyzed by two researchers (EC and LS). Workshops aimed to foster innovative thinking through collaborative brainstorming with stakeholders. Workshops were highly interactive, with stakeholders contributing directly to the Miro board in real-time. Workshops were also recorded: first, to allow researchers (EC and LS) to review the sessions and ensure all discussions were captured on the board; and second, to enable stakeholders who could not attend to watch the recording and contribute to the board. The board therefore remained accessible throughout the series, with the shared space facilitating ongoing engagement and allowing stakeholders to collectively build on progress made during each workshop. This continuous feedback ensured comprehensive and inclusive data collection.

The workshop series was conducted in three distinct stages–ideation, consolidation, and implementation–each aligning with steps in platform development as they occurred in real time. First, the ideation stage aimed to rapidly produce a working draft of the theory of change framework to inform and influence the product ideation strategy. Second, the consolidation stage aimed to create a simplified version of the framework to embed into product design. Third, the implementation stage aimed to produce an infographic that visually represented the finalized framework to share with digital mental health service staff, supporting their understanding of the platform.

#### 2.4.3 Dissemination design phase

An express media survey was employed using dscout (20) for youth to validate that the theory of change was understandable, that its key concepts resonated with them, and that the infographic effectively demonstrated the pathways to change. The survey was made available to 12 young people in the dscout pool aged 13 to 25, living in California and identifying as BIPOC.

Youth who chose to take part were introduced to the platform by narrative explanation and invited to view the infographic. The mixed-methods survey included qualitative open-ended questions and quantitative five-point scale questions, enabling youth to provide feedback on their first impression, visual appeal, clarity, and overall effectiveness of the infographic in conveying the platform’s mission and value for young people. A unique feature of the qualitative questions allowed youth to screenshot specific parts of the infographic, providing targeted feedback on areas they found unclear or particularly effective. Youth who completed the survey were compensated $10, in accordance with dscout’s policy (20).

### 2.5 Data analysis

#### 2.5.1 Qualitative insights

A rapid deductive qualitative analysis approach was applied to identify prominent themes from qualitative data collected during the three phases of work (**Figure 2**). Utilizing rapid analysis, as opposed to conventional content analysis, was essential to expedite sharing insights with stakeholders to hone in on the resulting outputs that would inform the next set of data collection (25, 26). Such an approach aligns with generative methods that prioritize iterative learning and co-creation (27). Analysis was conducted by two researchers for the insight generation (GS and EC), framework development (EC and LS), and dissemination design (RM and EC) phases.

For the insight generation phase, researchers summarized data from video transcripts and open-ended survey responses into four categories that reflected the broader goal of this phase: gathering youth feedback on the proof-of-concept app for the California Department of Health Care Services (DHCS) CYBHI grant proposal. These categories were: (1) DHCS testing criteria, (2) proof-of-concept app features, (3) impact and value to the user, and (4) product experience and functionality. Rapid analysis was conducted throughout the diary mission, with researchers identifying emerging themes within the four categories as each part of the mission progressed (see **eTable 1** for categories and corresponding themes). The thematic coding approach allowed for multiple categories to be applied to a single user expression, with codes assigned according to the nature of the entry. This ensured that insights remained closely aligned with the categories of themes needed to inform the next phase–defining the theory of change framework and guiding future iterations of the platform (25).

Within the framework development phase, rapid analysis was conducted immediately after each workshop during the workshop series. This involved: 1) reviewing workshop notes and recordings, 2) discussing theories and themes that emerged, and 3) considering how themes could be further clarified in subsequent workshops. This method produced immediate insights, enabling researchers to iterate and refine the workshop for subsequent groups by integrating emerging themes and adapting session activities based on the previous stakeholder discussions (25).

#### 2.5.2 Quantitative insights

Quantitative data were collected within the eight-day digital diary (insight generation phase) and express media survey (dissemination design phase). Data were analyzed using descriptive statistics suitable for nominal and ordinal data. Responses from single response and Likert-scale items were summarized by calculating frequency, median, and distribution range. Bar charts were created to highlight any strong positive or negative feedback patterns. By using a descriptive approach, we maintained an efficient, data-driven method to iteratively improve the infographic in line with youth feedback.

### 2.6 Ethical considerations

This work was conducted in accordance with the principles outlined in the Helsinki Declaration as revised in 2013, which sets ethical guidelines for research involving human participants. Ethical approval to conduct this work was not required, in accordance with the local legislation and institutional requirements, for service development and improvement, and usability testing activities.

## 3 Results

**Figure 4:**
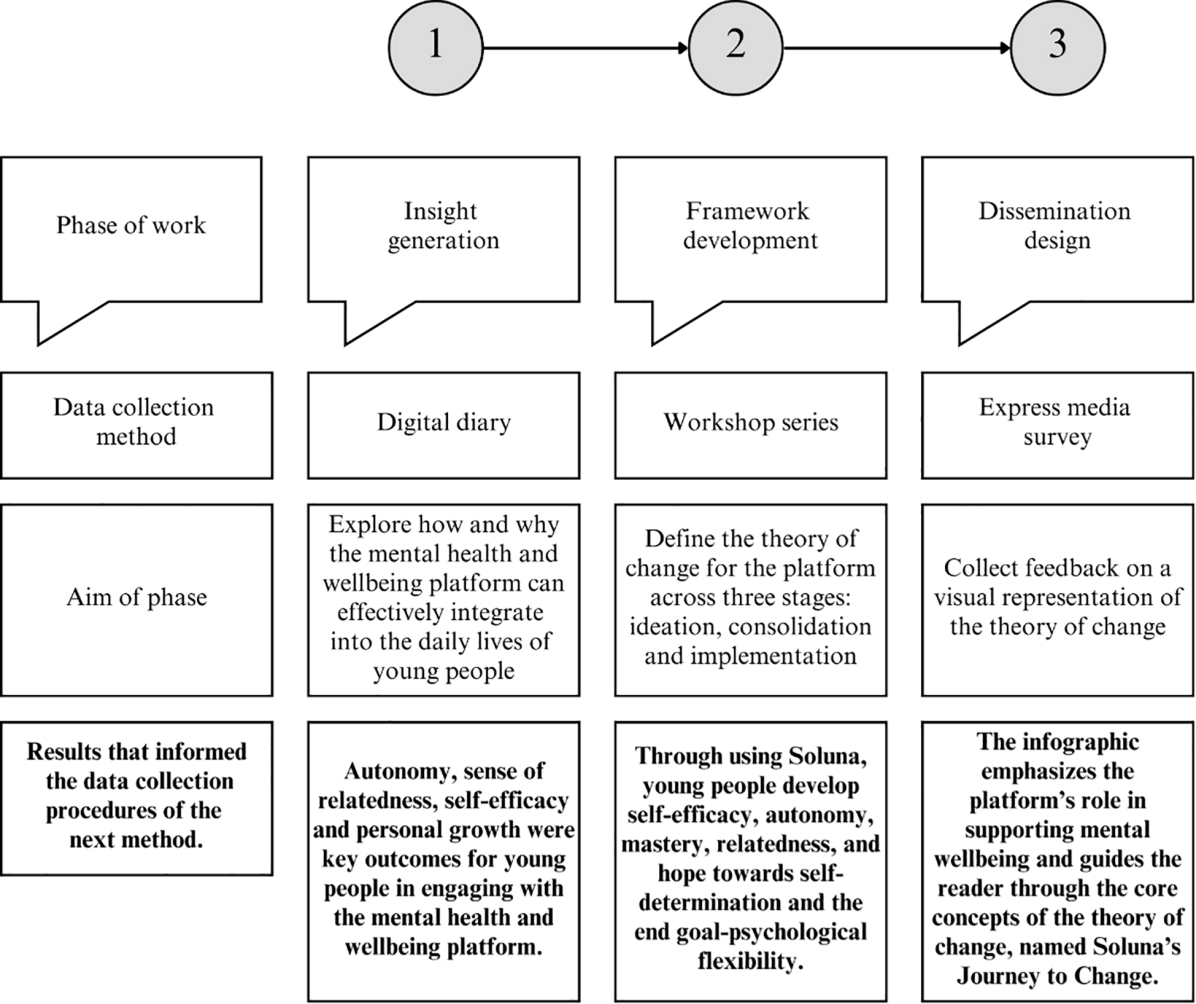
A flow chart outlining results from each data collection method that informed the data collection procedures of the next.

The collaborative, iterative process that combined youth, professional, and academic and industry expertise led to the development of the theory of change for the digital mental health and wellbeing platform. **Figure 4** presents key results from the three phases of work, which are discussed in detail in this section.

### 3.1 Insight generation phase

A total of 50 young people living in California completed the eight-day digital diary, providing real-time feedback as they used the proof-of-concept app. Among them, 4% were aged 13 to 16 and 96% were aged 17 to 25, and 56% identified as female, 44% as male, and 85% as BIPOC. Additionally, 75% said they were experiencing or had previously experienced anxiety, and 94% were currently navigating a moderate-to-major life transition. Examples of these transitions included shifting family or home dynamics, challenges at school or making friends, and illnesses or diagnoses.

#### 3.1.1 Integrating the app into their lives

At the end of the diary mission, 94% of youth reported that engaging with the app would be a positive addition to their daily or weekly routine. While using the proof-of-concept app, youth shared a variety of reasons for why they reached for it, and three key themes emerged: self-efficacy to accelerate personal growth, and building a positive (end-of-day) routine, and moments of need. These themes are discussed briefly below.

##### 3.1.1.1 Self-efficacy to accelerate personal growth

Youth used the app as a tool for personal growth. They revisited tools and activities that they found interesting, informative or helpful, took time for mindfulness and self-reflection, and explored the app out of curiosity to see how it might support their personal development and confidence in their own growth capabilities.

##### 3.1.1.2 Building a positive (end-of-day) routine

Feedback from youth indicated that they were most likely to use the app during the evening (55%) or right before bed (69%). They shared that this is the time they have more space to reflect on their day or themselves, are winding down, or have a heightened awareness of how they are interacting with technology and its impact on their mood and sleep. In addition, the app was often described as “calming”, with youth noting this as another reason for using it before bedtime.

##### 3.1.1.3 Moments of need

Youth also turned to the app in moments of need, using it as a distraction when bored, as a way to relax and unwind, or to seek guidance on handling a particular situation, such as dealing with relationships or coping with difficult emotions, like anxiety and stress.

#### 3.1.2 Motivations for seeking support through the app

From the digital diary, three key factors emerged as important to youth when seeking support through a mental health and wellbeing platform: autonomy and choice, sense of relatedness, and visual appeal. These are discussed briefly in turn below.

##### 3.1.2.1 Autonomy and choice

The ability to choose when and how to use the app was crucial for youth, particularly those focused on enhancing their self-esteem and self-determination. Results showed that 78% of users agreed or strongly agreed that they liked the app’s functionality. Insights indicated that this was likely because the app offered various ways to engage, including mixed media and interactive content. Autonomy and freedom to discover the app’s contents as they liked meant that the majority of young people found something helpful to them. Of all moments in the app, 87% were rated as useful or helpful by young people, rising to 93% among male respondents.

##### 3.1.2.2 Sense of relatedness

Youth reported that they were interested in what others who were like them were doing, or how they were coping with similar situations. Youth were seeking a safe sense of community, and wanted to connect with like-minded peers.

##### 3.1.2.3 Visual appeal

Visual design plays a critical role in the user experience in this context, as the way a digital experience looks, feels and moves, has a big impact on its perceived trustworthiness, value and relatability. The platform adopts a space-themed visual identity, incorporating constellation imagery and purple gradients designed to create a calming environment where users can explore their path to wellbeing. The top four terms used to describe the app were “calming”, “soothing”, “modern” and “relaxing”. Youth found the app’s design appealing, which opened the door to them to further relate to the app and its contents.

Overall, findings from the diary mission highlighted that autonomy, sense of relatedness, self-efficacy and personal growth were integral in setting the platform apart from other digital experiences. These insights were summarized and circulated to stakeholders ahead of the workshop series in the next phase of work to support theoretical discussions and identify how the platform’s features could foster these changes.

### 3.2 Framework development phase

The workshop series was conducted in three distinct stages–(2.1) ideation, (2.2) consolidation, and (2.3) implementation. The workshop series involved two academic and industry experts and 18 service staff, representing diverse teams including clinical, commercial, data science, data analytics, product, research, evaluation, and user research.

#### 3.2.1 Ideation stage

**Figure 5:**
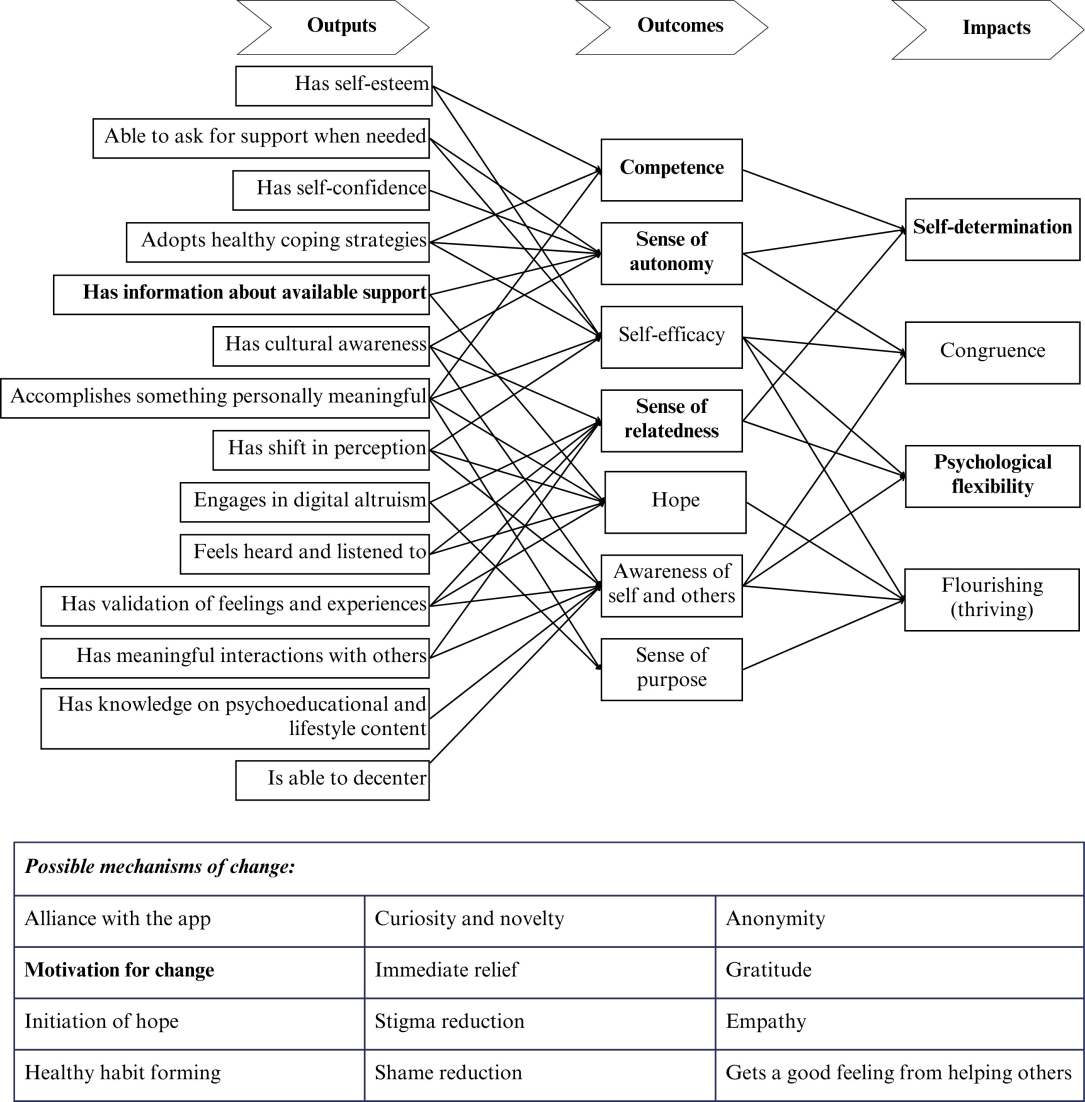
The working version of the theory of change for the platform, Soluna.

Five generative workshops were held during a one-week period. Workshop activities focused on three key areas: identifying outcomes and impact of the intervention, mapping pathways for behavior change, and aligning these pathways with existing behavioral change models, all within the context of the service’s humanistic value base (16, 17). During discussions, stakeholders contributed further theoretical perspectives in addition to the theories introduced at the initial workshop. As a result of activities at this stage, a working version of the theory of change framework for the platform was devised (**Figure 5**). The resulting framework (**Figure 5**) illustrates how constructs from established theories were embedded within the integrated design. The COM-B model (22) highlights the importance of capability and motivation in shaping behavior, self-determination theory (28, 29) emphasizes the role of autonomy, competence and relatedness, and Acceptance and Commitment Therapy (30) identifies psychological flexibility as its central mechanism of change. These constructs are shown in bold in **Figure 5** to visualize their integration within each stage of the framework.

The inductive exploration, alongside existing service values, led to the creation of a strengths-based, goals-focused theory of change model that is transcultural and transdiagnostic, designed to support a diverse youth population. As shown in **Figure 5**, four impacts were identified as the desired long-term changes for young people engaging with the app: self-determination, congruence, psychological flexibility, and flourishing. Seven key outcomes were then mapped to these impacts, including a sense of autonomy, relatedness, and self-efficacy, which emerged from youth feedback in the previous phase and were reinforced by stakeholders at this stage. The working version of the framework was shared with service staff, accompanied by a training video. This resource was designed to help staff understand and engage with the framework, enabling them to apply it directly during product ideation sessions.

#### 3.2.2 Consolidation stage

**Figure 6:**
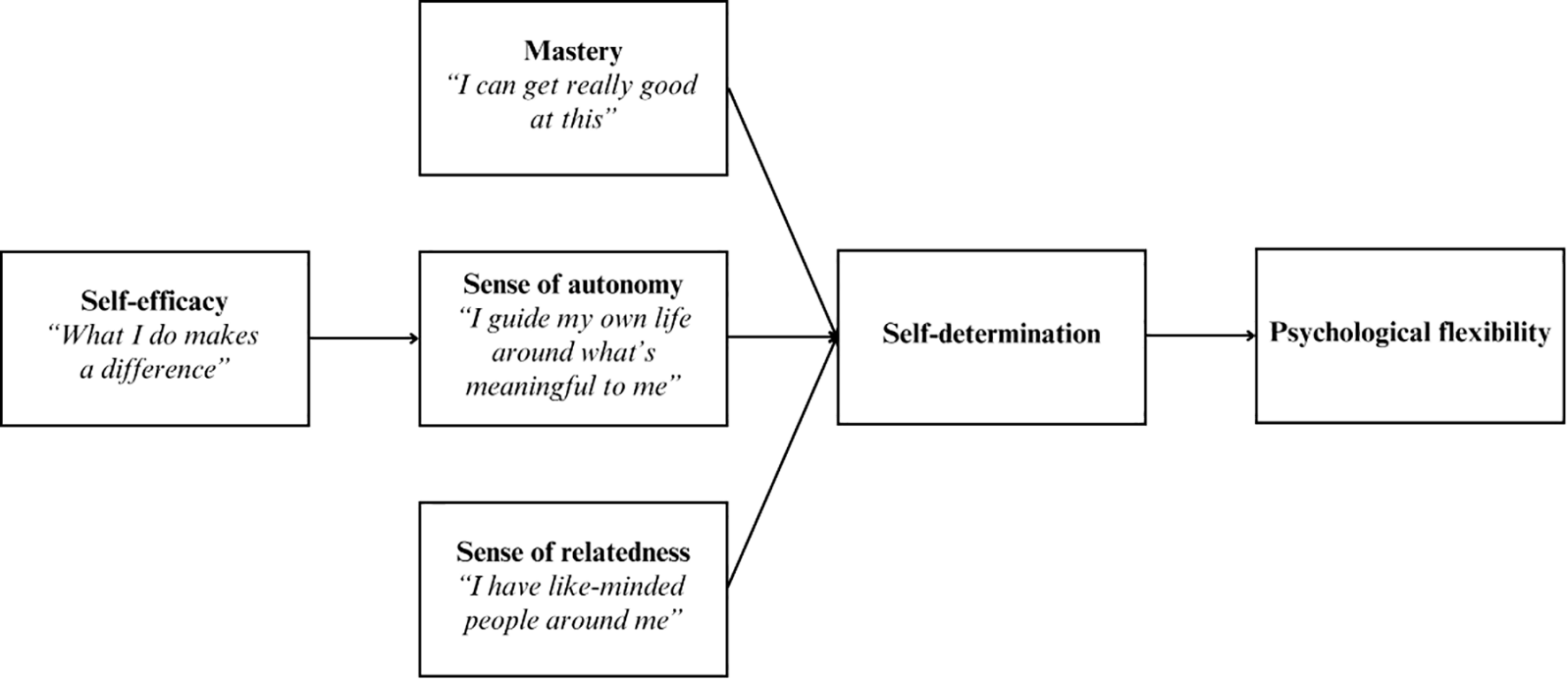
The grounded model that underpins the working theory of change for the platform, Soluna.

Two consolidation workshops were held to create a simplified version of the working theory of change that could be embedded into product design. Focused discussions aimed to synthesize data shown in **Figure 5**, incorporating asynchronous feedback received from stakeholders after the ideation stage. The resulting model defined the grounded theory that underpins the working theory of change (**Figure 6**).

The grounded model describes that the platform’s end goal is to help users build psychological flexibility, derived from theory underlying Acceptance and Commitment Therapy (30). Psychological flexibility is driven through self-determined change. Self-determination theory alone highlights three core needs: autonomy, mastery and relatedness (28, 29). Difficulties, such as loneliness, low mood or anxiety, arise when these needs are unmet. As shown in **Figure 6**, the grounded model illustrates how the mental health and wellbeing platform scaffolds behavior change by fostering self-efficacy, and promoting youth to make their own choices (autonomy) and enjoy learning new skills (mastery), while connecting them to others who share and support their values (relatedness). The grounded model was circulated to stakeholders, accompanied by a second training video, to help integrate the model into product design.

#### 3.2.3 Implementation stage

**Figure 7:**
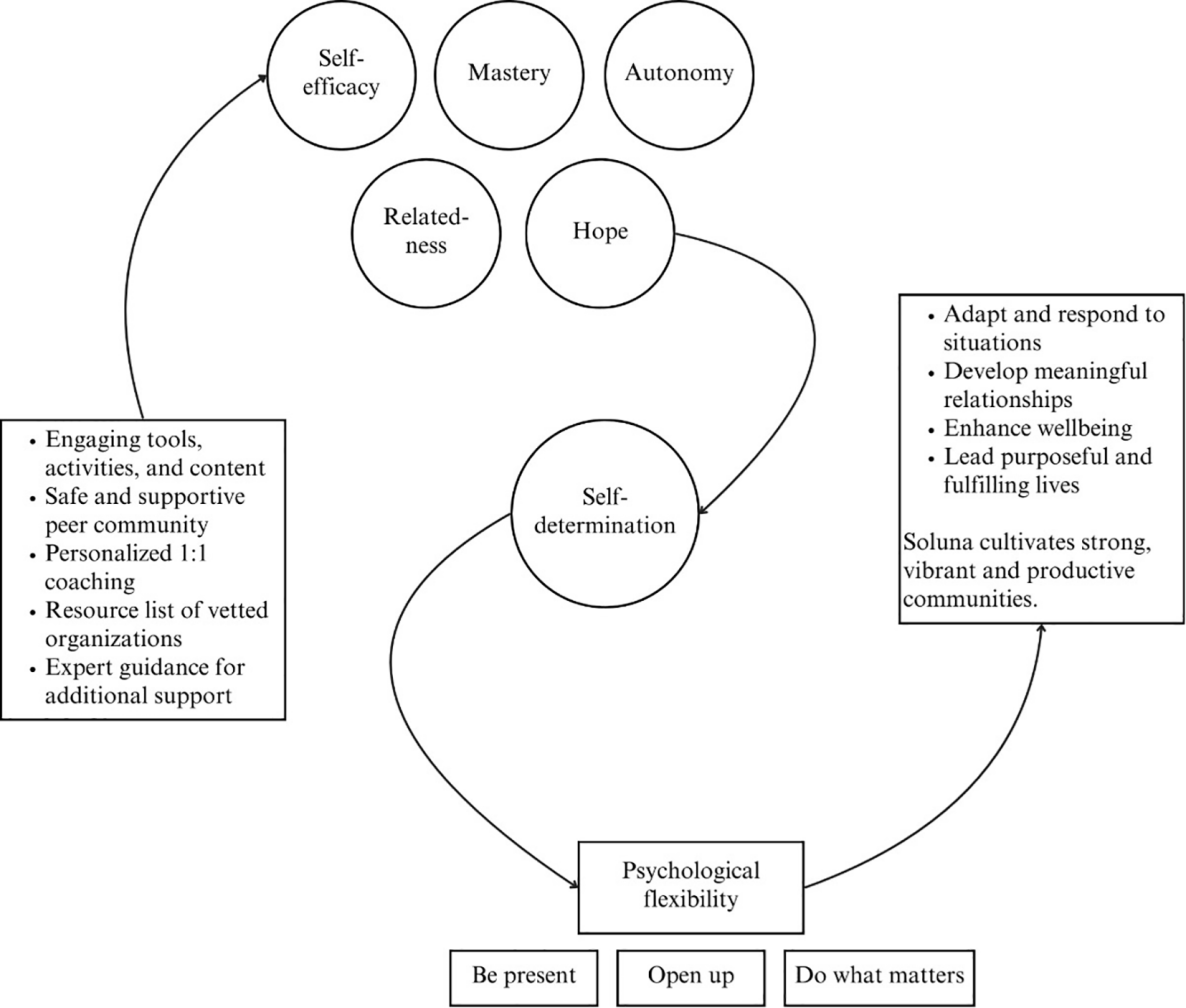
The design for the infographic that shows the logic connecting Soluna’s features to its user success metrics and impacts.

Two planning workshops were held to finalize the theory of change and design an infographic that was easily translatable, graphically descriptive, and showed the logic from the platform’s features to outcomes and impact. Discussions focused on summarizing the key service resources and platform features, and defining the long-term system impact which–aligned to the Community Capital Framework (31)– emphasizes sustained change at the community level. **Figure 7** presents the design of the infographic that represents the finalized theory of change.

As shown in **Figure 7**, the finalized theory of change outlines that the mental health and wellbeing platform, Soluna, provides individuals with a unique, three-pillar approach to developing skills: self-support, peer support, and professional support. Through using the platform, young people develop self-efficacy, autonomy, mastery, relatedness and hope towards self-determination and the end goal–psychological flexibility. This creates a positive ripple effect on their future, enhancing their wellbeing by helping them cope effectively with life’s challenges, build meaningful relationships, and lead purposeful, fulfilling lives. By achieving this for every young person, Soluna cultivates strong, vibrant and productive communities. The infographic that outlines this logic was shared with stakeholders, while an express media survey was created to collect feedback from youth in the subsequent dissemination design phase.

### 3.3 Dissemination design phase

Twelve young people from California completed the express media survey, designed to obtain feedback on the visual appeal and clarity of the infographic, as well as the title for the theory of change. Among these individuals, 25% were aged 13 to 16 and 75% were aged 17 to 25. The sample was ethnically diverse, with 50% identifying as Asian, 35% as Black or African American, and 14% as multiracial. Additionally, 42% identified as male and 58% as female.

#### 3.3.1 Visual appeal

The majority of respondents (67%) indicated that they “really liked” the visual design, while 33% found it acceptable, selecting that they “thought it looked okay”. No youth expressed dissatisfaction with the overall look of the poster. While most feedback was positive, several individuals suggested improvements, such as adjusting the color palette, simplifying the illustrations to avoid visual clutter, and shortening the text to improve readability. These modifications would collectively decrease the overall design complexity of the poster, thereby enhancing engagement.

#### 3.3.2 Clarity and understanding

Youth highlighted several areas where the theory of change infographic lacked clarity. Key feedback indicated that the target age group for the platform was not immediately clear. Additionally, some respondents were confused by the representation of concepts, such as mastery, self-efficacy, autonomy, relatedness, and hope, particularly in terms of how these elements were visually connected in the infographic. A few respondents mentioned that the introduction text for self-determination was unclear, and others noted that, while they recognized the importance of psychological flexibility, they were unsure of its meaning.

#### 3.3.3 Title preference

Young people were asked to select or suggest a title for the theory of change. The title ‘Journey to Change’ was favored by 50% of youth, making it the most popular choice. ‘Voyage to Change’ and ‘Pathway to Change’ followed, each preferred by 42% of respondents. In contrast, the ‘Theory of Change’ was favored by only 8% of youth and the ‘Engine for Change’ received no support.

#### 3.3.4 Revisions to the infographic

**Figure 8:**
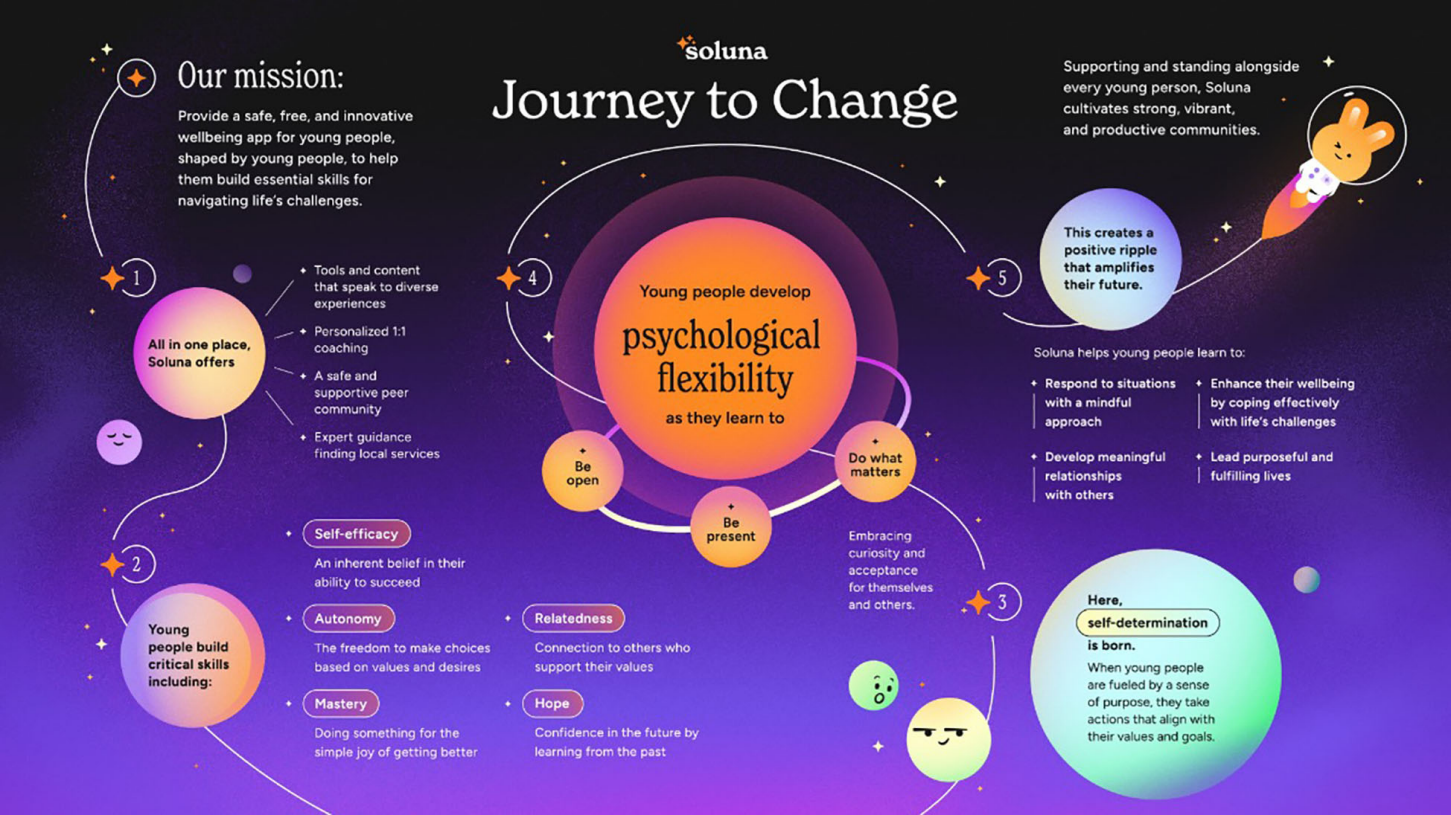
An infographic that represents Soluna’s Journey to Change.

In response to youth feedback, the infographic was revised to improve clarity and engagement. **Figure 8** depicts the finalized Journey to Change infographic, which now features a simplified design with clearer visual connections between concepts and a greater emphasis on making the platform’s purpose more accessible to young users.

The following revisions were made to the infographic as recommended by the youth:

- Streamlined and simplified the design and layout with a less-purple, modern aesthetic that is appealing to youth.
- The overall design clarified that the platform is a mental health and wellbeing platform and provided an improved visual guide through the steps of the Journey to Change.
- Revised the mission statement to provide concise and action-oriented language.
- Psychological flexibility is portrayed prominently, providing a visual cue of the significance of increasing psychological flexibility as a result of using the platform.
- Text changes to the three core parts of psychological flexibility, “be open”, “be present”, and “do what matters”, translated to actionable skills–a key element of the platform.

These revisions aimed to make the scientific concepts of the theory of change more accessible to staff and stakeholders. This became a resource for staff giving them a clear view of how the platform’s features and supports are designed to drive change and support mental wellbeing. The revisions also helped staff communicate the platform’s purpose and approach to stakeholders such as youth, teachers, and community partners.

## 4 Discussion

### 4.1 Principal results

This paper aimed to present the practical application of developing a theory of change for a digital mental health intervention, using a case illustration from Kooth Digital Health. A convergent mixed methods approach was used to develop a theory of change framework for the mental health and wellbeing platform, Soluna, across three phases of work: insight generation, framework development, and dissemination design. Three key stakeholder groups–youth from the platform’s target population, digital mental health service staff, and academic and industry experts–were consulted at different phases. This iterative process facilitated the development of a robust theory of change for the platform, and represented a pragmatic application of scientific methods tailored to real-world industry contexts, subsequently guiding the development of a user-centered intervention that was contextually relevant and effective. By understanding and applying these methods, developers and researchers can strengthen their ability to design digital mental health interventions that are both user-centered and effective.

For the insight generation phase, youth engaged in a digital diary to show how and why they might use the proof-of-concept app during a typical week. Rich multi-media feedback shared through the digital diary yielded important insights on the need for features that provide autonomy, foster relatedness, enhance self-efficacy, and support personal growth through skill-building and progress tracking. These findings provided a valuable foundation for designing a platform that closely aligns to the needs of its target audience.

For the framework development phase, cross-functional service staff members and academic and industry experts were consulted through a workshop series to define the theory of change framework, linking the platform’s inputs and activities to desired impact. Discussions defined a strengths-based, goal-focused approach for the intervention, designed to help users to make healthier, self-directed choices and to achieve meaningful change, in keeping with the humanistic psychology perspective that underpins the service (16, 17). During the workshop series, the theory of change was ideated, reviewed, and revised, reducing items to prioritize key concepts within the model, and ultimately outlining the pathways to building psychological flexibility. Concepts in the model closely align with existing behavioral change theories regarding youth mental wellbeing, such as self-determination theory (28, 29) and self-efficacy theory (32). The model also reflects third-generation behavioral and cognitive therapies in relation to Acceptance and Commitment Therapy to stimulate the necessary behavioral activation–a core element of modern applied psychology (30), collectively validating the approach to promoting meaningful behavior change.

For the dissemination design phase, youth provided feedback on the theory of change, presented through an infographic, to ensure that it was accessible to a broader audience. Based on youth feedback, the infographic was revised to enhance visualization and content clarity, emphasizing the platform’s role in supporting mental wellbeing and guiding readers through its core concepts. Feedback confirmed that, overall, the guiding outcome of psychological flexibility was one that young people also felt was important for improving mental wellbeing. This feedback was critical, as it validated that psychological flexibility resonated with their lived experiences and mental wellbeing goals. Their affirmation reinforced the importance of aligning the platform’s content and features with outcomes that are personally meaningful and relevant for young users, naming the model ‘Journey to Change’ to reflect this transformative process.

### 4.2 Overview of Soluna’s Journey to Change

The Journey to Change shares that Soluna’s mission is to provide a safe, free, and innovative mental health and wellbeing platform for young people, shaped by young people, to help them build critical skills for navigating life’s challenges. Soluna provides individuals with a unique, three-pillar approach to developing skills: self-support, peer support, and professional support. Through using Soluna, which drives behavioral activation, young people develop self-efficacy, autonomy, mastery, relatedness and hope towards self-determination, and psychological flexibility. Each feature of the platform directly correlates with the positive and intended outcomes of the Journey to Change model. For example, the platform’s pre-moderated peer community, which provides a safe and supportive space for young people to freely express their perspective, connects users with like-minded others. Thus, through the resultant activation of meaningful behaviors, users develop a sense of relatedness that fosters intrinsic motivation, which contributes to self-determination, and in turn, enhances psychological flexibility. In the longer term, psychological flexibility equips users with the skills to develop meaningful relationships, providing a supportive network that encourages them to lead purposeful and meaningful lives.

### 4.3 Strengths and limitations

The methodology adopted in this case illustration offers several notable strengths, including its collaborative approach and responsiveness to youth perspectives, which enhanced the relevance and applicability of the theory of change. These strengths are discussed together with reflections on the process and insights into its limitations. By considering both the advantages and constraints of this approach, we aim to provide a balanced perspective on its utility for future applications.

A key strength of the approach was the co-development of the integrated theory of change with three stakeholder groups: youth from the platform’s target population, cross-functional service staff, and academic and industry experts. The diverse composition of stakeholders involved at each stage of the process brought a range of perspectives that elicited rich discussions and contributed to a more comprehensive theory of change for the intervention (33). Youth were consulted at both the start and end of the development process described in this paper. Reflecting on this, an alternative approach would be to engage youth consistently throughout the process. For this to be effective, a co-design approach is recommended. This would involve collaborating with a representative group of young people as active partners from the outset. Through co-design, youth can develop a strong sense of ownership to the work, as well as benefit from the experience by gaining new skills, building relationships, and seeing their ideas influence decisions and direction. While youth involvement could be meaningful at every stage of developing a theory of change, it is important to carefully consider when and for what purpose their input is appropriate. Beyond the development process described in this paper, there is also significant opportunity to involve youth as part of an ongoing process to refine the theory of change for the intervention. Earlier work has recommended a formal revision process of the theory of change at key points in the intervention lifecycle, such as during implementation, evaluation and scaling stages (34). Establishing a youth-inclusive approach to the revision process would ensure that the intervention remains aligned with the evolving needs of youth, enhancing its relevance, effectiveness and overall impact.

A second strength of the outlined approach was the use of the convergent mixed methods design to inform the development of the theory of change. A mixed methods design is particularly valuable in complex fields like digital mental health as it enables triangulation of data from multiple methods, as well as different data sources (33). Using an iterative approach, this design allowed for the integration of data where the results from one method helped to inform the data collection procedures of another (19). In addition, within the framework development phase, data were analyzed using rapid qualitative analysis immediately after each session in the workshop series (25), providing real-time insights that were used to adapt activities for the next session. While effective in this context, rapid analysis approaches may not be appropriate if the project requires a high level of detail to meet its aims (25). Similarly, for the present work, an iterative, convergent mixed methods design was effective, allowing for the simultaneous collection and analysis of qualitative and quantitative data, but other methodologies could also be considered, such as exploratory sequential approaches. Exploratory sequential designs involve first collecting qualitative data; the insights of which are used to inform subsequent quantitative data collection. This alternative approach provides a structured way to build on qualitative insights and validate them through quantitative analysis (19).

For the current case illustration, two online platforms–dscout (20) and Miro (24)–were leveraged to facilitate interactive qualitative and quantitative data collection. Youth engagement utilized the diary mission and media survey methods available on the dscout platform (20). The diary mission allowed young people to naturally engage with the proof-of-concept app and share honest, unbiased and contextually-relevant feedback in an unmoderated setting. The express media survey gathered immediate, targeted responses to specific questions related to the theory of change infographic, enhancing the relevance and detail of the collected youth insights. The screenshot and video features further engaged young people, allowing them to provide specific and illustrative feedback (20). The visual collaboration platform, Miro (24), was leveraged for service staff and expert engagement. Miro allowed for real-time, creative collaboration among stakeholders, particularly suited for remote or hybrid teams, and facilitated ongoing opportunities for feedback. Additionally, its integration with tools like Zoom (23) enabled seamless workflow integration. However, it’s important to note that, while these online platforms are valuable, they do come with associated costs that should be considered as part of the work plan.

Another important strength is that the approach to the theory of change development remained flexible (34). This flexibility allowed for quick adaptation to unforeseen challenges and incorporated continuous feedback during the process, ensuring that the work could evolve in response to changing requirements or new insights. As an example, during the framework development phase, a few stakeholders were unable to attend workshop sessions at the last minute. In turn, additional sessions were held and session recordings were provided so those individuals could be part of discussions and contribute asynchronously. As another example, the consolidation stage of the workshop series was added into the work plan in response to stakeholder requests for a simplified version of the theory of change framework (**Figure 6**), which could be embedded into product design.

Though an adaptive approach was maintained, this also presents limitations. Workshops were resource-intensive, requiring significant human resources to plan, facilitate, consolidate and attend. To align with critical stages in the platform development process, fixed timelines were set for the ideation, consolidation and dissemination stages of the workshop series. As a result, though new workshops could be introduced, the same rapid timelines had to be adhered to, which increased pressure on the team. However, given the complexity of the intervention, it was critical for the overall approach to maximize inclusive participation, which ultimately strengthened the validity of the theory of change.

As referenced above, in this case illustration, the theory of change was established before platform development to serve as a roadmap for product design and strategy. Informational and training materials were produced at key milestones to foster a comprehensive understanding of the theory of change among service staff members and platform developers. On reflection, a significant challenge was giving these stakeholders the confidence to embed this knowledge into product design, particularly within a growing service. To address this, the need for theory of change advocates, or “champions” (34), who not only drive the theory of change development but provide ongoing support to stakeholders, was recognized. These advocates played a vital role in reinforcing the importance of the theory of change, bridging gaps in understanding, and demonstrating how the theory of change can inform the work of specific teams within the organization.

### 4.4 Future directions

A key contribution of this work is demonstrating how a theory of change framework can address a critical gap in digital mental health, where interventions are often evaluated by engagement and retention metrics without articulating the mechanisms through which change occurs (12). Developing a theory of change provides a structured framework that links the platform’s features to intended outcomes and impact. This transparency helps to ground the digital intervention in evidence-based practices, human-centered design, and iterative product development, while also creating the basis for an evaluation framework to demonstrate impact. Within Soluna, the Journey to Change framework operationalizes this approach. It will guide implementation strategies, such as performance monitoring, providing feedback to service staff on goals and progress, while future work will focus on demonstrating its value to young people who use the platform, which will also support successful implementation (35). Further, the Journey to Change provides a structured framework for evaluating the intervention’s effectiveness – from identifying indicators that track progress towards better individual outcomes to enhancing population health and delivering greater value for money in healthcare (36) – mediated by improvements in psychological flexibility.

### 4.5 Conclusions

Digital tools require a structured framework to ensure that they effectively serve their intended purpose. A theory of change framework outlines how an intervention’s activities lead to desired outcomes, fostering transparency, accountability, and evidence-informed decisions. Using a case illustration, this paper described the process of co-developing an integrated theory of change to guide the design and development of an intervention. A mixed methods approach involving three key stakeholder groups–target youth, cross-functional service staff and academic experts–was utilized. Together, their collaborative input was essential to shaping an intervention that is both user-centered and effective. In addition, this paper highlighted that this process should be iterative and flexible in its approach, as well as utilize interactive methods to engage stakeholders. Future work will use the theory of change framework to guide implementation and evaluation of the intervention, maximizing its impact in the digital landscape.

## Data Availability

The datasets presented in this article are not readily available because they are proprietary and subject to confidentiality restrictions. Requests to access the datasets should be directed to Kooth Digital Health; research@kooth.com.
