# Supplementary material for: Grounding digital mental health and wellbeing platform development in a theory of change: a convergent mixed methods approach
Source: Front Psychiatry. 2025 Oct 28;16:1637861. doi: 10.3389/fpsyt.2025.1637861 (PMC12603754; doi:10.3389/fpsyt.2025.1637861)
Supplement: Supplementary file 1 [file Table1.docx]

# **Supplementary Materials**

eTable 1 - Categories and corresponding themes used to analyze qualitative data during the insight generation phase.

| **Category** | **Themes** |
| --- | --- |
| California DHCS testing criteria | Relevance |
|  | Quality and usability |
|  | Functionality |
|  | Visual appeal |
|  | Ease of use |
| Proof-of-concept app features | Content - Format 1 |
|  | Content - Format 2 |
|  | Content - Format 3 |
|  | Tool 1 |
|  | Tool 2 |
|  | Tool 3 |
|  | Tool 4 |
|  | Tool 5 |
|  | Onboarding |
|  | Homepage |
|  | Library |
|  | Reach out or support |
| Impact and value to the user | Positive impact while using the app (e.g. felt calm in the moment) |
|  | Positive impact with additional constructive feedback (e.g. liked the visual and recommends adding sound) |
|  | Neutral or negative response while using the app (e.g. need for more relatable content) |
|  | In-app learnings/skills used in their day to day lives (e.g. new coping mechanism to help ground in moments of need) |
|  | Lived experience with mental health difficulties |
|  | Expectations of the app or its features prior to using it |
| Product experience and functionality | Positive feedback |
|  | User interface and graphics |
|  | User experience feedback |
|  | Content specific request |
|  | Feature improvement |
|  | Constructive criticism |
|  | Interaction design |
|  | Pre-app (new product request) |
|  | Post using app (new product request) |
